# ‘Like an infant … trying to run a marathon’: A longitudinal audio‐diary study exploring the transition from medical school to internship

**DOI:** 10.1111/medu.70221

**Published:** 2026-04-09

**Authors:** Stuart Redvers Pattinson, Hans Savelberg, Anique Atherley

**Affiliations:** ^1^ Unit for Undergraduate Medical Education (UUME), School of Clinical Medicine, Faculty of Health Sciences University of the Witwatersrand Johannesburg South Africa; ^2^ School of Health Professions Education (SHE) Maastricht University Maastricht The Netherlands; ^3^ Department of Nutrition and Movement Science, School of Health Professions Education (SHE), Faculty of Health, Medicine and Life Sciences Maastricht University Maastricht The Netherlands; ^4^ Director of Medical Education The University of the West Indies, Cave Hill Campus Bridgetown Barbados

## Abstract

**Introduction:**

The transition from student to doctor represents a challenging shift in identity and responsibility that many graduates find difficult to manage. To understand better how to support the transition to practice we need an exploration of graduates' experiences that does not see the transition as a single moment, but a continuous learning process. This study aimed to explore how medical students negotiate legitimate participation and professional identity formation (PIF) through time as they transitioned to internship in South Africa.

**Methods:**

We conducted longitudinal qualitative research using audio‐diaries and semi‐structured interviews to collect data from students over 7 months as they transitioned from medical school to several different health care institutions for internship. Twenty‐two students took part in entrance interviews, 20 collected audio‐diaries and 17 took part in exit interviews. Data were analysed using a narrative analysis approach, using communities of practice (CoP) theory as a sensitising–analytic framework.

**Results:**

We identified four dominant narrative plotlines in our data, revealing how legitimacy and PIF are constantly renegotiated through time. PIF faltered in medical school when students were excluded from hierarchical clinical teams, on graduation when they began to doubt their preparedness and in internship when participants were unable to demonstrate the competencies valued by CoPs within the demanding South African health care system. Professional identity was built when participants perceived themselves as being valued through their meaningful contributions to the shared enterprise of the CoP.

**Discussion:**

We call for a change in our framing of preparedness from ‘preparedness for practice’ to ‘preparedness for transition’, shifting our conceptualisation of preparedness towards equipping students with the resources they need for a complex, contextual, ongoing process rather than a moment in time. This requires clinical learning environments that legitimise trainees as peripheral participants, where learning is orientated towards gaining experience, cultivating professional identity and supporting individuals in developing the confidence, adaptability and resilience that will allow them to thrive as they negotiate the ongoing transition from student to doctor.

## Abstract 2

From ‘preparedness for practice’ to ‘preparedness for transition’ ‐ a longitudinal audio diary study exploring the challenging journey from student to doctor in South Africa. #MedEd

## 1 INTRODUCTION

Medical students entering the wards for the first time as internship doctors (interns) represents not just a new job title but a professional identity shift,^1^ leap in responsibility and potential source of significant anxiety.^2^ The transition from medical student to newly qualified junior doctor is often portrayed in the literature as inexorably challenging and a threat to graduate well‐being.^3^, ^4^ Concerns about high junior doctor burnout^5^ are significant due to the association with poorer patient outcomes, intent to leave the workforce and impaired trainee well‐being.^6^ Challenging transitions can, however, be a stimulus to learning if they are manageable.^7^ This highlights how important it is that students are prepared and supported in a way that sets them up for a transition where they can apply what they have learned, perform the job competently and confidently, feel a sense of belonging and continue to learn while maintaining their well‐being—thriving in their new role.^8^ Currently, however, many junior doctors seem to be simply surviving,^2^ underpinning a need to understand the complexities of the transition and better prepare and support trainees throughout their journey.

Attempts to mitigate the difficulty of the transition from student to doctor have tended focus on ‘preparedness for practice’; furnishing students with the clinical competence they need to do the job on day one of internship.^2^, ^9^, ^10^, ^11^, ^12^, ^13^, ^14^, ^15^ This can limit our understanding of the transition by viewing it as a moment in time rather than a continuous critically intensive learning period.^16^, ^17^, ^18^ Through longitudinal research, health professions educators can explore how medical trainee's experiences and contexts interplay and change through time,^19^ how they narrate the process of professional and personal growth^17^, ^20^, ^21^ and reveal what constructive and deconstructive challenges there are.^22^, ^23^ This allows for explicit undergraduate outcomes that are aimed at preparing students for the journey, rather than the destination.^23^, ^24^ In their 2021 narrative review of the literature on the transition from university to postgraduate medical training, Padley et al found very few original research studies that used longitudinal follow‐up, calling for more Longitudinal Qualitative Research (LQR).^2^ In recent years a number of LQR studies by Monrouxe et al.,^22^ Gordon et al.,^21^, ^23^ Ottrey et al.^25^, ^26^ and Liao et al.^27^ have deepened our understanding of transitions as ongoing, multiple and multidimensional,^23^ showing how professional identity is narratively constructed and renegotiated^27^ and how preparedness is not one unambiguous construct but varies over time.^25^, ^26^ There continues, however, to be a lack of LQR conducted in the global south.^28^, ^29^ Both Gordon et al.^23^ and Ottrey et al.^25^ who conducted LQR in the United Kingdom and Australia, respectively, have called for longitudinal research exploring transitions in underrepresented settings.

Context is important in HPE as learning is social and situated in the context in which it is applied.^30^ According to Lave and Wenger's communities of practice (CoP) theory, becoming a doctor is understood as a journey from legitimate peripheral participation through increased understanding towards full membership of the community.^30^, ^31^, ^32^ This involves developing both the repertoire of competencies that form the basis of legitimate practice within the community as well as a professional identity.^30^ In this study we consider professional identity formation (PIF) as progress towards thinking, acting, talking and feeling like a doctor, both through self‐definition and the definitions of oneself as presented by others.^33^ Development of a professional identity is paramount to becoming a successful doctor.^33^ Legitimacy is integral to this process and involves a sense of belonging and validation within a CoP derived from legitimate practice—displaying the knowledge and ways of being which are valued in that context.^34^, ^35^ CoP theory not only helps us conceptualise how learning and PIF happens in the clinical setting but also how it might be impeded, such as when the culture within a clinical team does not welcome peripheral participation. CoP theory has been critiqued recently for undertheorising power, and there has been a call for awareness of potential biases modelled by experts and recognition of when learners may not be experiencing equitable legitimate peripheral participation, particularly in global south contexts.^36^ In this study, context is not treated simply as background, but as shaping what counts as legitimate participation during transition. In South Africa, internship occurs in public‐sector settings with high service demand, resource constraints, uneven supervision and heavy responsibility for junior doctors, even recent graduates. The South African public health care system struggles to balance a quadruple burden of disease, infrastructural deficiencies and limited resources with providing training for medical students and internship doctors.^29^ In such settings, legitimacy may depend not only on what graduates know, but on whether they can cope, adapt, communicate and contribute under pressure. Studying transition in this context offers an exploration of how the basis on which preparedness, legitimacy and professional identity are negotiated may shift when trainees enter health care systems under strain.^29^

This study maps medical trainees' journey towards full participation at an early critically intensive learning period^18^—as they transition in their role from final‐year medical student to internship doctor. It is important to recognise that in this time they are still junior members of the CoPs they are joining (such as interprofessional teams in the wards, clinical departments and clinical units within a department—usually made up of consultants, registrars, medical officers, interns and students). It would be unrealistic to expect them to achieve full membership within the CoP in this early phase of what is an ongoing process, particularly considering their time‐limited placements and frequent rotations between departments. With that in mind, we focus on how legitimacy is negotiated by peripheral participants and how participants narrate their professional identity development through time. This study examines how legitimacy and professional identity are negotiated in a setting where transition occurs under systemic strain, allowing us to explore how context shapes the lived meaning of preparedness.

We aimed to answer the following question:

How do medical trainees negotiate legitimacy and professional identity through time as they transition from final‐year student to new internship doctor in South Africa?

## 2 METHODS

### 2.1 Study design

Underpinned by social constructionism, in which meaning is co‐constructed by people as they interact with the social world,^37^ we conducted longitudinal qualitative research, using audio‐diaries and semi‐structured interviews to collect data from medical trainees over 7 months as they transitioned from medical school to internship. Audio‐diaries allow for reflection‐in and reflection‐on experiences in real‐time and allow for a contemporaneous exploration of the participants' own construction of the transition as it evolves through time.^23^ Audio‐diaries, used alongside entrance and exit interviews, enable in‐depth longitudinal exploration, allowing us to consider how our participants narrate their evolving professional identities while empowering participants to make sense of those identities.^19^, ^38^, ^39^ Given the significance of sociocultural factors in understanding this important transition in our global south context, we used CoP theory^31^ as a sensitising–analytic frame in this study, using it to formulate our research questions and guide and orient our data analysis, but not imposing it on the data as a rigid pre‐defined coding structure.^40^

### 2.2 Setting and study population

Final‐year medical students were sampled from one medical school in South Africa based on convenience and access, as well the potentially wide range of experiences they would be able to share as they transitioned to a variety of different internship placements all over South Africa. This is a 6‐year medical programme with a graduate entry option into the third year of study. The first 4 years of the degree are largely medical school based, and the final 2 years consist of a rotation‐based clinical clerkship in health care facilities affiliated with the university. Graduates must then complete a mandatory 2‐year internship at an accredited public training facility. They rotate through Internal Medicine, Surgery, Paediatrics and Obstetrics and Gynaecology in their first year post‐qualification. Internship placements are available in all nine provinces in South Africa and are allocated by the Department of Health after an application process. In this clinical context, interns face high workloads, long work hours, low doctor‐to‐patient ratios and limited resources.^29^

All final‐year medical students at the university were emailed via official university channels with information about the study and invited to take part. All students who indicated their willingness to take part by a predetermined cut‐off date were included in the study. Twenty‐two of the 345 students in the class volunteered to take part. These 22 participants were placed in 13 different hospitals in six different provinces for internship by the National Department of Health.

### 2.3 Data collection

Data collection followed the steps set out in the AMEE guide on using audio‐diaries for research.^19^ The principal researcher (S. R. P.) conducted entrance interviews with all participants either in person or online using videoconferencing software depending on the participants' preference and availability. These took place between 30 September and 10 October 2024, at which time the students were starting their final clinical rotations of medical school and had just found out their internship placements. Entrance interviews were based on a semi‐structured interview guide (Data S1) that was developed by the researchers based on the research question, the theoretical frame and the relevant literature.^19^, ^23^, ^39^ Participants were sensitised to the audio‐diary process, a flexible schedule was set up for how participants would record and send their diary entries and any questions they had were answered. Participants were also given an Audio Diary Prompt Sheet (Data S2), which was discussed to sensitise them to the types of stories they could share. They were informed that they were free to reflect outside of the prompts if desired. The prompt sheet was then used as a scaffold for an introductory discussion on the basis of legitimate practice in medical school and participants' expectations for their transition to internship. Entrance interviews ranged from 16 to 36 minutes, averaging 26 minutes, resulting in a total of 9 hours and 32 minutes of transcribed data.

Of the 22 participants that took part in the entrance interviews, 20 recorded longitudinal audio‐diaries. Three participants recorded audio‐diaries for the first 3 months of the study while they were still in medical school but dropped out of the study once they started internship. Seventeen participants continued for the full duration of the study and took part in the exit interviews. A summary of the participants at different stages of the study is provided in Table 1 below. The longitudinal audio‐diary data collection took place from October 2024 to April 2025 (their last 3 months of medical school and first 4 months of internship). Participants were sent an email reminder once every 2 weeks. They were encouraged to record and send their entries whenever suited them to capture the in‐the‐moment experiences and there was no restriction on the length or number of diary entries. Participants recorded their audio‐diaries on their smartphones and emailed them to S. R. P., who acknowledged each entry received. This generated a body of data comprising 129 audio‐diary entries, ranging from 33 seconds to 28 minutes long (average 7 minutes), with an average of 7.6 entries per participant (ranging from 2 to 14 entries), resulting in a total of 14 hours and 28 minutes of transcribed audio‐diary data.

**TABLE 1:** Participant characteristics and involvement in the study.

Participants characteristics	Entrance interview (*n* = 22)	Longitudinal audio‐diaries (*n* = 20)	Exit interview (*n* = 17)
Gender			
Female	15	14	12
Male	7	6	5
Ethnicity			
Black	6	6	5
Indian/Asian	7	6	4
White	9	8	8
Internship placement—public hospital category^a^			
Regional	4	4	3
Tertiary	4	3	3
Central	14	13	11

^a^ Details regarding public hospital categories are provided in Data S4.

At the end of March 2025, participants were emailed a copy of their audio‐dairy transcript for their own record. This allowed them to review the transcript's accuracy and start reflecting on their experience of their transition and their study participation prior to the exit interviews. S. R. P. conducted exit interviews with the 17 participants online using videoconferencing software between 14 April and 20 May 2025. These were based on a semi‐structured interview guide (Data S3), the participant's transcripts and the audio‐diary prompt sheet (Data S2). During the exit interviews, participants debriefed their involvement and were given an opportunity to reflect on their data and the ‘long story’ of their transition, using their transcripts as prompts to discuss specific aspects of their transition experiences.^23^ Participants also provided feedback on their experience of using audio‐diaries and how this may have impacted their transition experience.^23^ Areas of uncertainty were clarified and participants were given the opportunity to add anything regarding their journey. Exit interviews ranged from 10 to 23 minutes, averaging 15 minutes, resulting in a total of 4 hours and 15 minutes of transcribed data. The audio‐diaries plus the entrance and exit interviews provided a total transcribed dataset of 192 789 words.

### 2.4 Data analysis

All audio data were transcribed and pseudonymised before being uploaded onto *Atlas.ti* for analysis. We used a narrative analysis^41^ approach to analyse our data, viewing the data using a situated lens and using CoP theory as a sensitising–analytic frame, aiming to understand how social and cultural contexts shape narrators' understandings of the world.^41^, ^42^ We viewed narratives as identity‐constructing sense‐making rather than transparent reports of experience.^20^, ^39^ S. R. P. and A. A. familiarised themselves with the data and then generated initial codes for the first three transcripts independently of each other. They then met to develop initial shared starting points for flexible coding, which evolved throughout the analysis through ongoing reflexive engagement. S. R. P. comprehensively coded the remaining transcripts and identified underlying narrative plotlines—key narrative patterns across all the participants,^20^, ^43^ which were then reviewed and refined by the whole research team. These narrative plotlines were analysed as they evolved through time,^28^ using common significant landmarks as identified by the participants (starting and completing their last medical school clinical rotation, preparing for and writing end of year exams, getting results, graduation, vacation, orientation and starting internship, their first call, the first few weeks/month of internship and rotating to a new department) to structure the longitudinal analysis. The stories of four participants are presented in the results section to illustrate the dominant narrative plotlines identified in our data, which are then summarised at the end of the results section. These participants chose their own pseudonyms, allowing them to present their stories with authenticity, capturing their personal experiences and cultural contexts while fostering trust and collaboration between participants and researchers.^44^ For context, Benjamin is a White male, Nafi an Indian male, Tendai a Black female and Grace a White female.

### 2.5 Ethical considerations

This work was carried out in accordance with the Declaration of Helsinki. The study was approved by the University of the Witwatersrand Human Research Ethics Committee (Protocol number: M240605) as well as the Maastricht University Faculty of Health, Medicine and Life Sciences Research Ethics Committee (Approval Number: FHML‐REC/2024/071). Written informed consent was obtained from all participants prior to the entrance interviews. It was emphasised that participation was voluntary and that the participants could, at any time, withdraw their participation.

### 2.6 Reflexivity

The research team consisted of the principal researcher (S. R. P.), who is pursuing a PhD in HPE, and two experienced educationalists and researchers (A. A. and H. S.). This study is part of a programme of research exploring preparedness for practice and the transition from student to doctor in South Africa, and builds on our previous work that identified a shift in the basis of achievement from medical school, where academic performance is rewarded, to internship, where personal and social competencies are valued.^45^, ^46^ We all brought our own ideas and experiences to the study, and this was discussed extensively within the team. The principal researcher (S. R. P.) is a lecturer involved in the third and fourth years of the undergraduate medical programme where the study took place and was familiar to the participants as one of their prior educators in their pre‐clinical years of study. He was not involved in their teaching or assessment as final year students or interns. S. R. P.'s interest in this area of study began when he noticed a pervasive trepidation among the undergraduate medical students regarding internship, a period of his own studies which he really enjoyed. His concern grew as he heard from recently graduated students that were really struggling with internship, motivating him further to explore this important transition to try to understand if there were ways students could be better prepared and supported.

A. A. is a physician and medical educator with a research interest in transitions during medical education. A. A. was born, trained and currently works in a small island developing state in the Caribbean, a context that sensitises her to issues of resource constraint in health care and relational learning. This positioning shaped her analytic attention towards developmental trajectories and meaning‐making during transitions. Having previously conducted longitudinal qualitative research on transitions and identity formation,^47^, ^48^ A. A. entered the study with pre‐existing theoretical lenses regarding the transformative potential of transitions, these preconceptions were treated as sensitising information as the current study progressed. Regular analytical discussions with the research team helped to interrogate where these assumptions might have extended or risked narrowing interpretations of the data in this current study. A. A. was not known to the participants.

H. S. is the developer of education in the health professions domain and teaches in bachelor and master programmes in Health Science, Biomedical Sciences and Medicine. As a teacher and curriculum developer, he noticed the challenge to develop programmes that drive students' intrinsic motivation to study and that have a transformative learning impact. As an educational scientist, he focuses on understanding design components that facilitate intrinsic motivation and transformative learning. This goes beyond knowledge transfer and learning the (medical) skills but requires developing a feeling of mastery and belonging. In this context, being qualified to perform the medical treatments is not sufficient; also, competencies in the social and personal domain are required. H. S. was not known to the participants.

## 3 RESULTS

This results section presents longitudinal narratives of four participants, chosen based on their deeply reflective and emotionally rich narratives, which allowed for analytic depth and provided compelling illustrations of the key underlying narrative plotlines in the data. These participants capture variation in affective stance, adaptation strategies and trajectories, but are not intended to be exhaustive representations of all participants. Each is an interpretive account of how they make sense of their experiences, negotiate legitimacy and construct their professional identity through time.

We then describe four dominant narrative plotlines that we identified across the majority of participants' narratives: (1) Students delegitimised and excluded from CoPs in medical school; (2) participants doubting their preparedness on graduation; (3) new interns struggling to cope, finding they are ill‐equipped to demonstrate the competencies valued by CoPs in a resource constrained setting and (4) developing a sense of belonging through valued contributions to the shared enterprise of the CoP. These are presented in Table 2 at the end of the Results section.

### 3.1 BENJAMIN: FROM EXCLUSION TO BELONGING—PARTICIPATION AS A PATHWAY TO PROFESSIONAL IDENTITY

Benjamin's story will illustrate how exclusion from clinical teams as a medical student can delay a student's sense of belonging and how supportive seniors and meaningful clinical involvement gradually enable students to negotiate legitimacy once they become an intern.

Towards the end of his last medical school rotation, Benjamin reflected on the ‘years of extortion and abuse and disregard’ that had ‘done a lot of damage’ as senior doctors were ‘egotistical, rude … just bitter, angry’. Many experiences were deeply negative, depending on how the clinical team viewed and treated medical students, often positioning him at the margins of the team and undermining his confidence and motivation. Yet moments of genuine inclusion revealed the transformative potential of participation. When seniors recognised that students had value and gave them a meaningful role to play in patient care, Benjamin felt engaged and motivated:

… felt a part of the team … not this like, silly student that doesn't know anything, I actually do have something to gain, but more importantly, to give … because of that, it made the late night calls, the overnight calls fun … we didn't mind being there, we wanted to be, we wanted to work hard.

These feelings of belonging were powerful as he shifted from observation to active participation, signalling an emergent sense of legitimacy rooted in mutual engagement and valued contribution towards a common goal.

As the end of the year approached, Benjamin was excited to finally put what he had learned into practice. But when graduation eventually arrived, it brought a complicated set of emotions associated with feelings of inadequacy and ‘imposter syndrome’. Benjamin was nervous about whether he knew enough and struggled to process the fact that he was now a doctor. He was ‘indifferent to the fact that I now have a title’ and was ‘still waiting for it to hit … still waiting to feel it and struggling to allow myself to feel proud’. Reconciling now having the title of ‘doctor’ with his own sense of identity was an uneasy task:

Every time someone refers to me as doctor, I don't know who they're talking about … very scary because of the responsibility that comes with that and the expectation of knowledge that I am assumed to have … I've literally just qualified.

The short break between graduation and internship was a blur of paperwork and ‘adulting’ tasks, figuring out the significant amount of admin involved in starting this new phase of life. Once internship began, Benjamin quickly recognised that he was still at ‘the bottom of the food chain’ and toxic behaviours persisted as interns were ‘abused … spoken down to and treated badly’. But the overall experience was more positive than he expected. He found supportive seniors, meaningful participation in patient care and a shared responsibility within the team helped him to negotiate legitimacy:

It is nice now to finally be treated as an individual and to be treated as a person of value … that can be a part of the team and can help. … one really fantastic part about internship is that you are very much part of the team, even if it means you're doing paperwork and you're doing the admin, you're still a part of the team.

Benjamin realised legitimacy was earned less through knowledge and more through getting the job done: ‘you're paid to do, you're not paid to think.’ This reframing helped him to grow quickly in confidence. He reflected on the fact that as students they get taught ‘a lot of super specialized things’ but not the core basics or common conditions that they needed to be able to manage as interns.

Three months into internship, nearing the end of the study, Benjamin looked back on a ‘tumultuous … very busy and uncertain’ journey, but it was rewarding. He credited his successful journey thus far to his mindset:

… you've got to have the right attitude; you've got to have the right outlook in order to succeed … you've got to really have the right resolve in order to make it through.

Still, at the end of the study, Benjamin admitted he was still shifting his professional identity, when asked if being a doctor has sunk in, he responded:

… every time students call me doctor, I'm like, call me Benjamin, I promise you I don't know anything …

Benjamin's story illustrates that the path towards legitimacy and professional identity was neither linear nor complete within the first months of internship. His narrative highlights how legitimacy was negotiated through inclusion and support—yet identity work and the feeling of ‘being a doctor’ remained ongoing.

### 3.2 NAFI: FROM SHOCK TO SELF‐CONFIDENCE—BUILDING LEGITIMACY THROUGH VALUED PARTICIPATION

Nafi's narrative illustrates the steep learning curve of early internship, showing how legitimacy develops, falters and is rebuilt. It shows how peer support and teamwork can enable interns to overcome emotional low points and grow in confidence as they perceive themselves as being valued by other members of the team.

In the final few weeks of medical school, Nafi's time and attention was focused on assessment. He was just pushing through, with energy and motivation waning:

I was just focusing on the finals and just getting through the end of medical school, just mustering up all the motivation that I could get at the end … I'm very excited for this next phase and to finally be done with medical school, to finally just, do the work that I've been training for and to not have to study.

He was critical of the extent to which how much you know formed the basis of achievement in medical school and shared his frustration with how much of their time in short clinical rotations was spent in tutorials preparing for theory assessments rather than participating and gaining clinical experience:

What's the point of coming to the hospital if you're just going to sit in a room all day … getting lectures basically?

Once he finally graduated, Nafi felt ready for what lay ahead. The reality of internship, however, was much more challenging than he expected. The first few weeks were the most difficult and stressful of his life and had him questioning whether he was able to continue in the role:

It was insane hey, those first two weeks. I really thought about leaving medicine, I won't even lie … that adjustment is insane … For me to be on call on my first official day was crazy, like, the amount of work that I had to do that … I didn't conceptualize before becoming an intern … the level of responsibility that was on me … I think I was just in a situation where it was a really busy night and, also, I didn't have the support that I thought I would have as an intern from the seniors, and I think it was just a culmination of a lot of factors that led to the most insane first call. And even the first week and the second week also were just crazy.

Nafi quickly realised that he had not really known what to expect from internship, and he recognised that he was not equipped with the repertoire of skills he needed to be valued as a member of the team. It was harder work, more responsibility and a bigger adjustment than anticipated. On his second call, he lost a patient he was looking after, a low point in his journey that shook his confidence:

… that responsibility was on me, because I was the intern on call. And one of the doctors came and debriefed me, and I asked her this question; ‘all I want to know is how do I get back to work right now? Like, I feel like I'm in a state of shock.’ … it's traumatic to lose a patient.

As the first month of internship progressed, Nafi found himself in a draining cycle of work and recovering from work and more exhausted than he'd ever been, the work–life balance he had hoped for had not materialised.

Nafi found that one of the hardest parts of finding his place was dealing with hierarchy and conflict. He had a tough time figuring out power dynamics, working relationships, setting boundaries and dealing with blame and criticism from seniors who appeared to have become apathetic and jaded. He reflected on how he hoped not to go down that same path himself:

… we can sit here and say doctors are exhausted and burnt out and jaded and that's why they so mean and that's why they so frustrated … I'm frustrated, I am very frustrated, and I'm overwhelmed and I'm working so hard and I'm exhausted … I get barely any sleep, I've been working every single weekend and it's only January, but I don't want to be a mean person, like, I'm not going to come out of this being a mean person just because I'm tired and I'm overwhelmed and I'm overworked … that's my intention and I'm going to try and stick to it.

For Nafi, internship did get better with time, thanks largely to a community of interns with a common purpose that looked out for each other and shared the workload. By recognising the importance of teamwork and communication and improving his own efficiency and organisation, Nafi turned a corner and started to grow in self‐confidence as he began to perceive himself as being seen as a valued member of the team. Being pushed to his limits showed him that he was much more capable than he had ever thought.

Things have improved, I have gotten faster and more efficient. I'm more confident, you know, I can speak up for myself and advocate for my patient a lot more, which is a huge turning point for me.

Nafi's story highlights how legitimacy is earned through demonstrating the industry and teamwork that is critical to the community's joint enterprise of patient care within a resource‐constrained setting. Like Benjamin, Nafi struggled with belonging, but his mechanism of adaptation was collective rather than individual, underscoring the importance of teamwork and effective communication skills in helping interns navigate the early shock of responsibility.

### 3.3 TENDAI: FROM OVERWHELMED TO FINDING JOY – RESILIENCE IN THE FACE OF ADVERSITY

Tendai's journey illuminates the ways students can find themselves unprepared for the transition to internship and the impact this has on their well‐being. Through a strong support system and reframing their perspectives and mindset through reflection, interns can persevere in the face of significant contextual challenges.

As much as Tendai wanted to spend the last few weeks of medical school focusing on preparing for internship, between the pressure to pass and end of year fatigue after ‘six years of sleepless nights, all‐nighters, tears shed and sacrifice’, she found it difficult to look past just overcoming the last few assessments. Once those final exams were all finally passed, Tendai started to look forward to a period of independence and personal growth as an intern. Yet she experienced some discomfort with the recognition of what it means to have become a doctor, struggling with celebrating her achievements and finding the attention a little bit daunting. Part of her still felt ill‐equipped for what lay ahead.

As internship started, Tendai ‘felt like an infant trying to take their first steps, but not even first steps, trying to run a marathon’. As internship progressed, Tendai struggled with the long hours and lack of sleep as fatigue started to feel like a ‘constant companion’. The anxiety of handling emergencies alone and a lack of confidence in her capabilities started to take their toll. Overwhelmed with the sheer volume of work and an endless list of tasks, Tendai could not believe that this is what all those years of studying had led to:

You're doing your best to get through your to‐do list, but there is resistance from all sides, from patients, a broken system, or from your own fatigue. The hospital is understocked and understaffed. Medications are expired, machines don't work, patient care is compromised, and the blame often lands on the doctors … You're running on two hours of sleep, trying to remember something you last studied two years ago in a two‐week block, and you can't help but feel … deeply inadequate. You keep going because you have to; tasks don't disappear just because you're tired. And somehow, there's beauty in that resilience. But it is not an easy task. Internship has been one of the hardest things I've ever done. It has stretched me in ways I still don't have the words for. Maybe in a year or two, I'll be able to look back and appreciate the growth, but right now? It's raw. No one could've prepared me for the transition from student to doctor. People warned me—but some things you have to live to understand. The long hours, the unshakeable self‐doubt, the anxiety that follows your every decision, the leap from student to being the person that people turn to in an emergency, is massive. I'm not just observing anymore… And there's no lecture, no single conversation that could've made me feel more ready for this moment.

In the face of these challenges, Tendai persevered, relying on the support from her community of family, friends and peers. She found she was able to find joy in connecting with others; moments of teamwork, solidarity and shared laughs gave her a sense of belonging. As the study ended, she was beginning to enjoy herself and live in the moment, using reflection as a powerful tool to bring about a major turning point in her mindset:

One hard truth I've had to confront is the lie we tell ourselves in medicine: ‘After this block of exams, things will be better.’ ‘After surgery, life will be easier.’ ‘After internship, it will calm down.’ But this thinking just keeps you in a cycle of stress, always chasing a finish line that keeps moving. What has been more helpful is learning to take things one day at a time, allowing myself to exist beyond medicine.

Looking back, Tendai could not believe how much she had been through and overcome, recognising the start of internship as a period of ‘immense growth’—both growth in her confidence as a doctor and also personal growth as a human. Reflecting on the skills she needed to overcome challenges that were not only academic, but also physical and emotional, Tendai urged the university to treat students more as valued individuals, encouraging personal development and equipping them with the skills they need for internship:

I really wish that medical school would place more emphasis on the human side of this journey. Beyond having the ability to regurgitate every line in a textbook, what I have found to be more important is being adaptable and maintaining a willingness to learn.

Tendai's narrative, like Nafi's, demonstrates the consequences of a junior doctor finding themselves unprepared with the repertoire of competencies needed for legitimate practice within the South African public health care context. Tendai realised that what counts is not just knowledge and clinical skills, but a broad set of personal and social competencies that allow students and interns to reflect, adapt and learn as they face new challenges.

### 3.4 GRACE: FROM SURVIVING TO THRIVING—CULTIVATING PROFESSIONAL IDENTIFY THROUGH MEANINGFUL CONTRIBUTION

Grace's reflections on her internship experiences highlight the ways students could be better prepared for the challenging transition to practice. Through rediscovering a passion for patient care, students and interns can start thinking, acting and feeling like a doctor by meaningfully contributing to the mutual enterprise of the team.

With a few months to go in medical school, Grace struggled to navigate the tension she saw between preparing for assessments and preparing for internship. To her, the curriculum did not seem well aligned with what she expected from the junior doctor role and the assessments did not seem a good indicator of readiness:

The marks are what's going to get you through at the end of the day, so that feeling of juggling between; do I put more emphasis on what's going to make me a better doctor or more emphasis on what will allow me to become a doctor in the first place?

As her time as a student was ending, Grace was both nervous and excited for internship. Her nerves came from a fear of failure, anxiety over handling emergencies on her own and not having had enough opportunity to put what she did know into practice:

… the challenge is … those feelings of incompetence, you know … you can't believe that there's only a few more weeks left … and then soon it's your responsibility, and the onus is on you to take care of patients and people will expect … when they call for the doctor, that's you. And there is a lot of trepidation around that, where you've just theoretically learnt things versus putting them into practise.

The start of internship turned out to be a huge jump for Grace. She was thrown in the deep end and the hard work, long hours and very little time off resulted in her starting to work herself ‘into the ground’ by the end of her first month. Her work–life balance was nowhere near normal. She started to recognise where her priorities should have been as a student:

… getting cum laude and 90% is not the most important thing … for me that's been really eye opening coming into internship like, everything that is made internship pleasant has come out of, not because I studied hard … but every pleasantness has come from where I took the time to stay at the hospital and learn as much as I could.

Grace realised that what would have made the biggest difference in her being better prepared for internship was if she had been welcomed into clinical teams as a student instead of feeling in the way or having didactic teaching take priority over experience:

As a student, I didn't realise how important it was to stick with a team, work with the team … I understand it's difficult, like when you have tutorials and stuff, to go along with everything the team is doing, but it's so important because you miss out on so much and there is so much happening all the time and I feel like a lot of it I never realised.

The biggest turning point for Grace came a few months into internship when she started learning how to take better care of her own well‐being, looking after her sleep, diet and exercise in a way that allowed her to be the best version of herself. Much of her enjoyment was derived from recognising how much she was growing and learning as a doctor and the rewarding feeling of having a meaningful impact on a patient's life:

I've also been managing patients from admission to discharge and the satisfaction of looking a patient's mom in the face and saying, ‘you're going home’ and just, like, that smile and then thanking you, has just been really beautiful and really reignited my passion … I think that the highs are very high and I know the lows are very low, but that feeling of knowing that you did everything you could and this patient that came in and was so sick and not okay and now is okay and is going home and just … the gratefulness and the feeling that I can do this, it's just been a really, really lovely feeling.

Grace's narrative illustrates how missed opportunities to welcome students as legitimate members of clinical teams can result in a transition to internship marred by feelings of inadequacy and trepidation. For Grace, thriving as an intern was about actively maintaining a healthy work–life balance and, like Benjamin, her sense of identity was rooted in being able to connect with patients and contribute to the shared goal of patient care.

### 3.5 DOMINANT NARRATIVE PLOTLINES

We identified four dominant narrative plotlines in how participants negotiated legitimacy and professional identity, related to three distinctive phases in the transition from medical school to internship. These are presented in Table 2 below. These plotlines were nonlinear, often overlapped, involved stops and starts, detours, reversals and evolved uniquely for each participant.^28^

**TABLE 2:** Dominant narrative plotlines.

Plotline	Phase	Legitimacy	Professional identity
Students marginalised or excluded from hierarchical clinical teams as priority is given to preparing for and performance in upcoming high stakes assessments.	Final clinical rotation in medical school.	Legitimate peripheral participation is impeded within the CoP as the basis of achievement resides in demonstrations of knowledge in assessments rather than gaining experience and contributing as part of the team.	Students are not recognised as adding value in the clinical space and are made to feel in the way, preventing them from developing a sense of belonging and gaining confidence.
Self‐doubt and discomfort with the new title of ‘doctor,’ anxiety and nervousness for the start of internship.	Graduation.	Tension between the legitimacy granted by the university through graduation with uncertainty over their preparedness for the expectations of the role.	Trying to reconcile being qualified as a doctor without feeling like a doctor.
Overwhelmed by the expectations and responsibility of working in a broken system with jaded and apathetic seniors, leading to fatigue, poor work–life balance and burnout.	Start of internship.	Inability to demonstrate the repertoire of skills valued by a CoP situated within a high intensity, resource limited context results in delegitimisation.	Sense of identity falters when participants' inability to cope with their new role leads to doubt about whether they are cut out to be a doctor.
Growing confidence and competence through adaptability, reflection, resilience, willingness to learn, strong support systems and building positive work relationships.	Start of internship.	Gaining legitimacy through recognising and displaying the competencies that form the basis of achievement in the CoP such as efficiency, reliability, teamwork and communication skills.	Professional identity development through meaningful contribution the shared goal of the team, defining themselves through being valued by other members of the CoP.

*Note*: Table 2 summarises the four dominant plotlines identified at different stages in the participant's narratives and the role each of these had in negotiating legitimacy and professional identity as they transitioned from final‐year medical student to internship doctor.

Abbreviation: CoP, communities of practice.

## 4 DISCUSSION

This study explored how final‐year medical students narrate their transition from medical school to internship in South Africa. Our findings show that legitimacy during this transition is not achieved once, but continually renegotiated as trainees attempt to demonstrate the competencies required to function within hierarchical clinical teams, demanding workloads and resource‐constrained health systems. These findings highlight how legitimacy and PIF unfold within the specific conditions trainees encounter during early clinical practice.

The process towards thinking, talking acting and feeling like a doctor for our participants was complex, nonlinear and remained ongoing throughout our study. Using CoP theory as a sensitising analytic‐frame, we were able to show that participants' professional identity was built whenever they felt a sense of legitimacy, perceiving themselves as being valued by their CoP through recognition of their meaningful contributions to the shared enterprise of the team.^30^, ^33^ Legitimacy was, however, not easily achieved and regularly faltered. In medical school, legitimate peripheral participation was often impeded by students being excluded in the clinical setting by hierarchical teams that did not provide them with the trust and support required to develop a sense of belonging.^30^, ^32^ Graduation was coloured by feelings of uncertainty and self‐doubt as participants grappled with their identities and questioned their preparedness for internship, which had been based on academic performance rather than experience in the context in which they would now be expected to work.^49^ These concerns turned out to be well founded as legitimacy continued to be renegotiated when participants started internship and realised they were not equipped with the repertoire of competencies required to be recognised as valued members of a CoP situated in the demanding South African public health care context.^30^, ^36^ Their professional identity and confidence was shaken as they became overwhelmed and began to doubt whether they had what it takes to be a doctor in a context where ‘valued competencies’ differ from medical school and reflect the ability to function within an overburdened health care system.^45^, ^46^ Navigating this tumultuous journey towards becoming a doctor required more than just clinical knowledge and skills, but adaptability, resilience, willingness to learn and a strong support system.

Monrouxe argues that even if medical students learn all the knowledge and skills required to graduate, they will struggle to practice with confidence and be successful as doctors until they have developed their professional identity,^20^, ^33^ and by uncovering important experiences and contextual factors that hinder legitimate peripheral participation, our findings add to our understanding of the ways professional identity development might be impeded. Our findings are in keeping with other studies, which describe the transition from trainee to trained doctor as complex, relational, multiple and ongoing,^23^ leading to a process of PIF that is narratively constructed, dynamic, constantly transforming and continually renegotiated.^27^ Although our findings echo prior work showing transition as ongoing and emotionally challenging,^23^, ^26^ they deepen that literature by showing how, in our setting, legitimacy depends not only on knowledge but on the ability to function within a strained health system through efficiency, adaptability, teamwork and endurance.^29^, ^45^, ^46^ Although researchers have called for recalibration of stakeholders' expectations of what new graduates should be able to do on graduation,^22^ our findings suggest this needs to be balanced with preparing students for the reality of a context where legitimacy, and therefore their ability to successfully transition to their new role as a junior doctor, is unevenly granted and comes from having the competencies required to cope within a ‘broken’ system that is slow to change.^45^, ^46^

We join Ottrey^25^, ^26^ and Monrouxe^22^ in calling for clarity of what exactly we mean when we talk about preparedness and problematising check‐box approaches. There are many ways of conceptualising preparedness, particularly within different contexts, and a lack of shared understanding can result in misplaced expectations.^22^, ^26^ This work extends existing literature on preparedness by showing that what ‘counts’ during transition is partly produced by the service context trainees enter. Taking this discussion forward, we call for a conceptual shift in our framing of preparedness from ‘preparedness for practice’ to ‘preparedness for transition’. This reframing positions preparedness as readiness for a complex, ongoing process rather than a discrete event. ‘Preparedness for practice’ often emphasises endpoint capability—for example, the ability to diagnose and treat a patient under supervision on day one—whereas ‘preparedness for transition’ may better consider that in settings where graduates enter strained systems with substantial responsibility and variable supervision, preparedness cannot be understood only as readiness to perform predefined clinical tasks under supervision. It must also include readiness to negotiate legitimacy, identity, relationships and uncertainty across changing communities and conditions. Educators should aim to equip graduates not only with clinical knowledge and skills needed for practice but also with the reflexivity required to adapt positively to multiple tranitions,^2^, ^23^ allowing them to feel ready and resilient in the face of change.^23^ This shifts our focus beyond what trainees *know* towards *who they are*. Instead of seeking smooth transitions, challenges can be seen as opportunities for valuable personal and professional development^16^ where transformational experiences are lived and reflected on.^48^ Importantly, it should also serve as a reminder that guiding and supporting trainees does not stop at the end of medical school but is the continued responsibility of all CoPs throughout this ongoing transition, including those in the internship setting.^23^

### 4.1 Recommendations for practice

As learning and engaging in practice are part of the same process, and professional identity is negotiated through personal experiences and how we participate,^33^, ^50^ CoPs need to be intentional about welcoming and valuing students in the clinical space, giving them the opportunity to develop trust, confidence and competence as legitimate members of our clinical teams.^51^, ^52^ This requires that we examine the social and cultural structures embedded in practice to ensure that the attitudes and behaviours within our learning environments enable legitimate participation.^50^, ^52^ Social structures tend to reproduce themselves so, without intervention, the hierarchical power relationships, conflict and exclusion experienced by our participants are likely to continue.^30^ Faculty development is essential and seniors need to be encouraged and supported in providing supervision, role modelling, mentoring and feedback to students and interns.^2^, ^30^

Students need to be provided with defined roles within clinical teams and given the opportunity to actively participate in patient care,^51^ allowing them to feel useful,^7^ cultivate their professional identities and acquire new knowledge, skills and experiences out of interest, not just because it will be assessed.^3^ Students need authentic longitudinal clinical attachments without the constant pressure from high stakes theory assessments or a predominance of didactic teaching, allowing them to come to understand the complex, flawed system in which they will soon be working and actively work on the competencies that will enable them to inhabit the intern role in a way that is valued.^7^, ^16^, ^49^

Our participants received a striking lack of support for their complex transition to practice, and there is a pressing need to correct this problematic situation with a deliberate, planned approach to supporting individuals throughout medical school and internship.^23^ The types of competencies needed to be ‘prepared for transition’ rather than ‘prepared for practice,’ such as adaptability, resilience and self‐regulated learning can be learned and developed through authentic real‐life experiences with opportunities for graded responsibility and the promotion of self‐reflection.^2^, ^17^ Explicitly communicating the value of and then proactively coaching skills such as teamwork, reflection, reflexivity, effective self‐care, maintaining a work–life balance and time management is likely to help graduates learn to deal with stressful change and adapt positively as they make the challenging transition to practice.^16^, ^17^, ^53^, ^54^, ^55^

### 4.2 Methodological strengths and challenges

To our knowledge, this is the first study exploring the student to internship doctor transition using LQR in South Africa. Our study has several strengths and challenges that should be taken into consideration when interpreting our findings. Our participants all graduated from one South African university only, so our findings may lack transferability to different educational and clinical contexts. As our diverse group of participants transitioned to internship, however, they were able to share experiences from a variety of institutions, improving transferability of our findings within the South African and other similar underrepresented contexts.^2^ Our use of longitudinal qualitative data in exploring transitions into clinical practice in the underrepresented global south context does fill important methodological and contextual gaps in the literature.^29^ We collected a substantial and rich dataset and had a low attrition rate, generating a sample with sufficient information power given the focused study aim, the specific sample, the use of established theory, high dialogue and audio‐diary quality and the longitudinal analysis strategy.^56^, ^57^ Our low attrition rate was achieved through flexible data collection, regular communication and maintaining researcher continuity. S. R. P. was able to build rapport with the participants, as he was known to them as one of their previous lecturers, and tap into their keenness to help the next generation of graduates. The longitudinal nature of this study allowed us to explore participants' narratives through time and, while some of the audio‐diary entries were retrospective reflections over longer periods, many were current thoughts and feelings, and therefore were less reliant on distant recall than retrospective interviews conducted long after events.^26^ Our use of narrative portraits allowed us to honour our participants' powerful stories.

Our participants were still in the early stages of their journey by the end of the study, and our findings do not represent later stages of the ongoing student to doctor transition, nor did we explore differences in experiences depending on where participants were placed for their internship, which presents an opportunity for further research. This research also did not specifically investigate how issues of race and gender impacted how legitimacy and professional identity development were narrated, which is an important area for future research, particularly in the South African context with its legacy of colonialism and Apartheid.^29^, ^35^, ^36^

## 5 CONCLUSION

This LQR study explored how PIF is narrated through time as final‐year medical students transition into internship, revealing how legitimacy is constantly renegotiated based on the culture and values of the communities of practice they encounter within the challenging South African public health care system. We call for a change in our framing of preparedness from ‘preparedness for practice’ to ‘preparedness for transition’, shifting our conceptualisation of preparedness towards equipping students with the resources they need for a complex, contextual, ongoing process rather than a moment in time. This requires that we re‐evaluate the culture and values within our clinical learning environments to ensure students are legitimised as peripheral participants and that learning is orientated towards gaining experience, cultivating professional identity and supporting and guiding students in developing the confidence, adaptability and resilience that will allow them to thrive as they negotiate the ongoing transition from student to doctor.

## AUTHOR CONTRIBUTIONS

**Stuart Redvers Pattinson:** Conceptualization; investigation; writing—original draft; methodology; writing—review and editing; project administration; data curation; formal analysis; validation; resources; visualization. **Hans Savelberg:** Conceptualization; validation; resources; supervision; writing—review and editing. **Anique Atherley:** Conceptualization; methodology; formal analysis; validation; resources; supervision; writing—review and editing.

## CONFLICT OF INTEREST STATEMENT

The authors have no competing interests to declare.

## Supporting information


**Data S1.** Annexure A: Entrance Interview Schedule.

**Data S2.** Annexure B: Audio Diary Prompt Sheet.

**Data S3.** Annexure C: Exit Interview Schedule.

**Data S4.** Annexure D: Categories of Public Hospitals in South Africa.

## Data Availability

The data that support the findings of this study are available on request from the corresponding author. The data are not publicly available due to privacy and ethical restrictions.
