# Supplementary material for: ‘Like an infant … trying to run a marathon’: A longitudinal audio‐diary study exploring the transition from medical school to internship
Source: Med Educ. 2026 Apr 9;60(10):1130–42. doi: 10.1111/medu.70221 (PMC13569273; doi:10.1111/medu.70221)
Supplement: Supplementary file 1 [file MEDU-60-1130-s001.docx]

**Annexure D**

**Health Professions Council of South Africa (HPCSA)**

**Medical and Dental Professions Board Handbook on Internship Training**

**Guidelines for Interns, Accredited Facilities and Health Authorities^1^**

**Levels of Health Care: Categories of Public Hospitals**

**District Hospitals**

District hospitals are categorised into small, medium and large district hospitals with the following number of beds. Small district hospitals with no less than fifty (50) beds and no more than one hundred and fifty (150) beds. Medium size district hospitals with more than one hundred and fifty (150) beds and no more than three hundred (300) beds. Large district hospitals with no less than three hundred (300) beds and no more than six hundred (600) beds.

A district hospital must –

a. Serve a defined population within a health district and support primary health care.
b. Provide a district hospital package of care on a 24-hour basis.
c. Have general practitioners and clinical nurse practitioners providing health services.
d. Provide services that include in-patient and ambulatory health services as well as emergency health services.
e. A district hospital receives outreach and support from general specialists based at regional hospitals.

A district hospital may only provide the following specialist services –

a. Paediatric health services.
b. Obstetrics and Gynaecology.
c. Internal medicine.
d. General Surgery.

**Regional Hospitals**

A regional hospital must, on a 24-hour basis, provide –

a. Health services in the fields of Internal Medicine, Paediatrics, Obstetrics and Gynaecology, and General
b. Surgery.
c. Health services in at least one of the following specialities –
d. Orthopaedic Surgery
e. Psychiatry
f. Anaesthetiology
g. Diagnostic Radiology
h. Trauma and emergency services
i. Short term ventilation in a critical care unit

Services to a defined, regional drainage population, limited to provincial boundaries and receives referrals from several district hospitals. A regional hospital receives outreach and support from tertiary hospitals. A regional hospital has between four hundred (400) and eight hundred (800) beds.

**Tertiary Hospitals**

A tertiary hospital –

a. Provides specialist level services provided by regional hospitals.
b. Provides sub-specialities of specialities referred to in paragraph (a).
c. Provides intensive care services under the supervision of a specialist intensivist.
d. Receives referrals from regional hospitals not limited to provincial boundaries.
e. Has between four hundred (400) and eight hundred (800) beds.

**Central Hospitals**

A central hospital –

a. Must provide tertiary hospital services and central referral services and may provide national referral services.
b. Must provide training of health care providers.
c. Must conduct research.
d. Receives patients referred to it from more than one province.
e. Must be attached to a medical school as the main teaching platform.
f. Must have a maximum of one thousand two hundred (1200) beds.
g. Central referral services are provided in highly specialised units, require unique, highly skilled and scarce personnel and at a small number of sites nationwide.

National referral services –

a. Refer to super-specialised national referral units.
b. Represents extremely specialised and expensive services (e.g. heart and lung transplant, bone marrow transplant, liver transplant, cochlear implants).

References

1.	Health Professions Council of South Africa Medical and Dental Professions Board Handbook on Internship Training Guidelines for Interns, Accredited Facilities and Health Authorities. Pretoria, 2025 edition.
