# Supplementary material for: ‘Like an infant … trying to run a marathon’: A longitudinal audio‐diary study exploring the transition from medical school to internship
Source: Med Educ. 2026 Apr 9;60(10):1130–42. doi: 10.1111/medu.70221 (PMC13569273; doi:10.1111/medu.70221)
Supplement: Supplementary file 2 [file MEDU-60-1130-s002.docx]

**Annexure C – Exit Interview Schedule**

Introduction:

- Informed consent, privacy and confidentiality (as per the information and consent form)
- Request to record the session.

Purpose of the exit interview

- Debrief on the experience of taking part in the study
- Obtain participant feedback
- Reflect on the participant’s data
- Clarify areas of uncertainty
- Opportunity to add any additional insights
- Reflect on the ‘long story’ of their transition from student to internship doctor

Questions:

- How your experience of taking part in the study? What was it like recording the audio diary entries?
- Did taking part on the study and recording the audio diary have an impact on your transition to internship?
- Do you have any feedback for us researchers regarding your experience of the study as a participant?
- What were the thoughts and feelings you experienced as you reviewed and reflected on your diary entries over the last six/seven months?
  - Clarification of any areas of uncertainty regarding the data
- Is there anything you would like to add that you missed in your audio diary reflections?
- Who would you describe your overall experience of the transition from student to doctor?
- Possible additional prompts
  - Has your experience of internship so far matched your expectations?
  - What challenges did you face?
  - What competencies do you need to succeed in internship?
  - How well prepared were you? Are there ways in which you could have been better prepared?
  - What helps and what hinders a successful transition to practice?

Thanks
