# Supplementary material for: ‘Like an infant … trying to run a marathon’: A longitudinal audio‐diary study exploring the transition from medical school to internship
Source: Med Educ. 2026 Apr 9;60(10):1130–42. doi: 10.1111/medu.70221 (PMC13569273; doi:10.1111/medu.70221)
Supplement: Supplementary file 3 [file MEDU-60-1130-s003.docx]

**Annexure B – Audio Diary Prompt Sheet**

Below are some things to think about when recording your audio diary entries. These are just a guide; please don’t feel limited by these prompts or feel like you need to answer all of them. Please feel free to be flexible and reflect on any aspect of the transition from student to doctor you would like to.

Please record a reflection that answers some or all the following questions:

*During your last few months at medical school:*

- How is medical school going? What have been the recent stand out experiences?
- What does it mean to be ‘doing well’ or succeeding at medical school?
- What specific challenges are you currently facing or have recently faced?
- What are your expectations regarding the student to doctor transition? How well prepared do you feel?
- Is there any specific experience that stands out as it relates to your expectations or preparation for internship?
- What experiences have you had recently that you think will help or hinder your transition to internship?
- How do you anticipate the transition will go?
- What do you expect internship will be like?
- What skills will you need to succeed in internship?

*During the first few months of internship*

- How is internship going? What have been the recent stand out experiences?
- How would you describe the transition from student to doctor?
- Has your experience of internship so far matched your expectations? What have been the surprises?
- What specific challenges are you currently facing or have recently faced?
- What competencies/attributes/skills do you need to succeed in internship? What makes a ‘good’ intern?
- How well prepared were you? What are the specific parts of the job you felt well or not well prepared for?
- Are there specific ways in which you could have been better prepared?
- What has helped or hindered your transition to internship?
- What advice would you give a current medical student to help them be better prepared?
- What advice would you give to the medical school to better prepare graduates for internship?

Important Reminders:

- Please try record and send an audio diary entry at least once every two weeks
- Participation is voluntary and you can withdraw at any time without any penalty
- If you find taking part in this study or recording an audio diary entry distressing in any way, please contact family or friends or consult your GP, mental health care provider, family LifeLine South Africa (0861 322 322) or South African Depression and Anxiety Group helpline (0800 567 567)

Thank you very much for participating in this study.
