# Supplementary material for: ‘Like an infant … trying to run a marathon’: A longitudinal audio‐diary study exploring the transition from medical school to internship
Source: Med Educ. 2026 Apr 9;60(10):1130–42. doi: 10.1111/medu.70221 (PMC13569273; doi:10.1111/medu.70221)
Supplement: Supplementary file 4 [file MEDU-60-1130-s004.docx]

**Annexure A – Entrance Interview Schedule**

Introduction:

- Informed consent, privacy and confidentiality (as per the participant information and consent form)
- Request to record the session.

Information regarding the study:

- Aim
  - To explore the lived experience of the transition from student to internship doctor.
- Purpose of the entrance interview
  - Provide information regarding the scope and process of the research study
  - Answer participant questions
  - Set up schedule for recording and sending audio diary entries
  - Discuss the distress protocol
  - Discuss the types of stories to share in their reflections
- Study design and requirements of participants
  - Longitudinal study involving recording of audio diaries over a six-month period
  - Aiming for once every two weeks but this is flexible
  - Recorded on smart phone and emailed to principal researcher
  - Online exit interviews

Questions regarding the study:

- Do you have any questions about what participating in the study involves or how the study will work?
- Do you have a smart phone, and will you be able to record and send audio diary entries?
- Will a schedule of every two weeks work for you, or would you like to set up an alternative schedule?
- Would you be happy to receive reminders? What time and day of the week would suit you for reminders the communication method would suit you?
- Do you know what to do if you find participating in the study distressing in any way?
- Will you be happy to take part in an online exit interview via Microsoft Teams?
- Are you still happy to participate in the study?

Questions to sensitise participant to study aims and types of stories to share:

- How is medical school going?
- What competencies do you need succeed at medical school?
- What challenges have you faced?
- What are your perceptions of the student to intern transition?
- How well prepared do you feel for internship? How do you anticipate the transition will go?
- What do you expect internship will be like? What skills will you need to succeed?

Facilitator summary and conclusion.

- Summary of research schedule going forward
- Reminder of voluntary nature of participation and that they can withdraw at any time without any penalty

Thanks
